# The US elections as a determinant of global health

**DOI:** 10.1016/j.lana.2024.100884

**Published:** 2024-09-11

**Authors:** Muhammad Jawad Noon, Xinshu She, Benjamin Mason Meier

**Affiliations:** a Department of Economics, University of Göttingen, Göttingen, Germany; b Harvard T.H. Chan School of Public Health, Harvard University, Boston, MA, USA; c Department of Pediatrics, Stanford University, CA, USA; d Department of Health Policy & Management, University of North Carolina at Chapel Hill, NC, USA

Global health is crucial for the United States, preventing health threats and enhancing economic growth. US investments in global governance, workforce training, and clinical research improve preparedness for health emergencies and support international stability. Addressing shared health challenges in a globalising world requires global health collaboration. This approach not only aligns with humanitarian values but also supports US economic and security interests. The 2024 US elections will determine the direction of the country's engagement with global health.

Global health initiatives have long received bipartisan support in the US. Following World War II, President Truman advocated for the advancement of health under the United Nations (UN) and supported the formation of the World Health Organization (WHO).^1^ President Eisenhower expanded this support, seeking to build a healthier post-war world, and amid Cold War challenges, President Kennedy solidified US engagement through the 1961 Foreign Assistance Act and launch of the United States Agency for International Development (USAID). President Johnson extended these efforts by establishing the Fogarty International Center at the National Institutes of Health (NIH) to facilitate global health research and training and supported WHO's smallpox eradication program. President Carter strengthened US commitment by backing WHO's 1978 Declaration of Alma-Ata to build public health capacity in low- and -middle income countries.

While President Reagan limited support to WHO and politicised global health initiatives in the 1980s, the US recommitted to global health by the 1990s. President Clinton reinforced multilateral global health partnerships through the UN Millennium Development Goals and the Global Alliance for Vaccines and Immunization. President Bush developed the President's Emergency Plan for AIDS Relief (PEPFAR) in 2003, which treated over 20 million people across 54 countries.^2^ President Obama furthered this commitment through the 2015 establishment of the UN Sustainable Development Goals and the Paris Agreement to address climate change. These efforts across administrations underscored the importance of sustained US investment in global health.^3^

In a stark departure from this long-standing commitment, the Trump/Pence administration's “America First” agenda marked a shift toward populist nationalism. Global health was marginalised in US foreign policy, neglecting public health, humanitarian imperatives, and global governance. The expansion of the “Mexico City Policy,” which prohibits US foreign aid recipients from engaging in abortion-related activities, jeopardised vital global programs addressing HIV/AIDS, Zika, and maternal health.^4^ The Trump/Pence administration's immigration policies separated families, violated rights, and harmed health. The administration's stance also threatened multilateral initiatives like the Global Health Security Agenda (GHSA) and bilateral PEPFAR support for HIV/AIDS treatment. Trump/Pence environmental policy rollbacks and withdrawal from the Paris Agreement undermined international climate negotiations.

The Trump/Pence administration's skepticism toward international institutions challenged global health governance at a critical moment. Early in the COVID-19 pandemic, the administration sought to withdraw the US from WHO membership, disrupting the international response. Domestically, the administration politicised public health efforts, opposed masking and physical distancing, and interfered with public health agencies, weakening collective action against COVID-19.^5^ Despite these missteps, the Trump/Pence administration launched Operation Warp Speed (OWS), investing $18 billion in expedited vaccine development and averting preventable deaths in the US.^6^ Yet, the administration did not join worldwide initiatives to ensure equitable vaccine access globally.

Reversing many of these policies, as framed in Table 1, the Biden/Harris administration began with sweeping Executive Orders on COVID-19, climate change, and economic security, seeking to reestablish international cooperation and evidence-based decision-making. The administration undertook significant bilateral and multilateral steps to improve global health, including resuming WHO membership and rejoining the Paris Agreement. The Inflation Reduction Act marked the most significant US investment in reducing carbon emissions. While at times undercut by specific new fossil fuel drilling approvals, this legislation has directed billions in clean energy and environmental health initiatives.^7^

**Table 1:** Divergent US approaches to global health per federal administrations.

Trump/Pence administration	Biden/Harris administration
• WHO Withdrawal • Interfering Politically in Public Health Decision-making • Rejected climate science and withdrew from Paris Climate Agreement • Abandoned COVAX • Cut UNFPA funding and reintroduced the Mexico City Policy • Introduced migrant family separation policy	• WHO Support • Supporting Independent Public Health Policy-making • Rejoined Paris Climate Agreement and introduced Inflation Reduction Act • Joined COVAX • Reinstated UNFPA funding and rescinded the Mexico City Policy • Reunited affected migrant families and ended Title 42

Moreover, the Biden/Harris administration revitalised the U.S. commitment to reproductive health by revoking the “Mexico City Policy,” restoring funding to the UN Population Fund (UNFPA) and supporting global women's health programs.^8^ The administration also ended Title 42 and started humanising asylum processing at the US-Mexico border. Although the US continued to hoard COVID-19 vaccines unnecessarily, it joined the COVID-19 Vaccines Global Access (COVAX) partnership and pledged $4 billion to promote vaccine access worldwide. Supporting these policy shifts, the Biden/Harris administration has become a collaborative partner in global health law reforms under WHO, including amendments to the International Health Regulations and the development of a new Pandemic Agreement.^9^

In the 2024 US presidential election, the future of global health is at a critical juncture. A potential Trump/Vance administration has signalled a return to isolationist policies, including a likely withdrawal from the WHO and the Paris Agreement,^10^^,^^11^ as well as plans to cut funding for international humanitarian and reproductive health programs.^12^^,^^13^ Undermining international climate action, the Trump/Vance campaign has rejected climate science and proposed policies to deregulate fossil fuel production. By contrast, the Harris/Walz campaign advocates for international cooperation, reproductive health, and climate change mitigation. In her current role as Vice President, Harris has been central to climate change policies, announcing a $3 billion pledge to the UN Green Climate Fund,^14^ and the Harris/Walz campaign seeks to extend existing environmental policies of the Biden/Harris administration. Harris has emphasised the importance of upholding international humanitarian law, which is paramount for protecting both health and human rights, as the US rejoined the UN Human Rights Council, reversing the Trump-era withdrawal. Nonetheless, the Biden/Harris administration has faced criticism for the escalating humanitarian crisis in Gaza, exposing glaring inconsistencies in its commitment to human rights and international law.

The 2024 US elections present a choice between two contrasting visions for global health. As the world continues to grapple with emerging and ongoing health challenges, the direction taken by the next president will impact global governance, international cooperation, and health policies. The outcome of this election will be pivotal for the future of global health.

## Contributors

MJN: conceptualization, investigation, writing—original draft, writing—review & editing; XS: investigation, writing—review & editing; BMM: conceptualization, supervision, writing—review & editing.

## Declaration of interests

The authors are members of the Advocacy and Communications Committee of the Consortium of Universities for Global Health (CUGH). This is an unpaid, voluntary position.
